# Synthesis of Isoxazole and 1,2,3-Triazole Isoindole Derivatives via Silver- and Copper-Catalyzed 1,3-Dipolar Cycloaddition Reactions †

**DOI:** 10.3390/molecules21030307

**Published:** 2016-03-04

**Authors:** Mohamed Mehdi Rammah, Wafa Gati, Hasan Mtiraoui, Mohamed El Baker Rammah, Kabula Ciamala, Michael Knorr, Yoann Rousselin, Marek M. Kubicki

**Affiliations:** 1 Laboratory of Heterocyclic Chemistry Natural Products and Reactivity/LCHPNR, Department of Chemistry, Faculty of Science, University of Monastir, 5000 Monastir, Tunisia; wafa.gati@yahoo.fr (W.G.); mtiraoui1hasan@gmail.com (H.M.); rammahmbaker@gmail.com (M.E.B.R.); 2 Institut UTINAM—UMR CNRS 6213, Université de Franche-Comté, 16 Route de Gray, 25030 Besançon, France; ciamalak@yahoo.fr; 3 Institut de Chimie Moléculaire—UMR CNRS 6302, Université de Bourgogne, 21078 Dijon, France; Yoann.Rousselin@u-bourgogne.fr (Y.R.); Marek.Kubicki@u-bourgogne.fr (M.M.K.)

**Keywords:** [3+2] dipolar cycloaddition, arylnitrile oxides, arylazides, isoxazole, 1,2,3-triazoles

## Abstract

The CuI- or Ag_2_CO_3_-catalyzed [3+2] cycloaddition of propargyl-substituted dihydroisoindolin-1-one (**3**) with arylnitrile oxides **1a**–**d** (Ar = Ph, *p*-MeC_6_H_4_, *p*-MeOC_6_H_4_, *p*-ClC_6_H_4_) produces in good yields novel 3,5-disubstituted isoxazoles **4** of the ethyl-2-benzyl-3-oxo-1-((3-arylisoxazol-5yl)methyl)-2,3-dihydro-1*H*-isoindole-1-carboxylate type. With aryl azides **2a**–**d** (Ar = Ph, *p*-MeC_6_H_4_, *p*-OMeC_6_H_4_, *p*-ClC_6_H_4_), a series of 1,4-disubstituted 1,2,3-triazoles **6** (ethyl-2-benzyl-3-oxo-1-((1-aryl-1*H*-1,2,3-triazol-4-yl)methyl)-2,3-dihydro-1*H*-isoindole-1-carboxylates) was obtained. The reactions proceed in a regioselective manner affording exclusively racemic adducts **4** and **6**. Compared to the uncatalyzed cycloaddition, the yields are significantly improved in the presence of CuI as catalyst, without alteration of the selectivity. The regio- and stereochemistry of the cycloadducts has been corroborated by an X-ray diffraction study of **4a**, and in the case of **6a** by XH-correlation and HMBC spectra.

## 1. Introduction

One of the research activities of our laboratory in the domain of heterocyclic chemistry deals with the 1,3-dipolar cycloaddition of nitrile oxides and azides as dipoles across the double or triple bonds of dipolarophiles [1,2,3]. A very recent example is the 1,3-dipolar cycloaddition of aryl nitrile oxides to the allyl group of ethyl 1-allyl-2-benzyl-3-oxo-2,3-dihydro-1*H*-isoindole-1-carboxylate providing a series of isoxazolines [4]. This versatile strategy for the synthesis of heterocyclic compound is more and more used in materials chemistry, drug discovery, and chemical biology [5,6,7]. Since the pioneering work of Huisgen, the contributions of Sharpless to “click chemistry” have given an additional impetus for the advancement of cycloaddition reactions [5,6,7,8,9,10,11,12,13,14,15] which are nowadays a trusted tool in targeted synthesis, especially for those involving the construction of heterocyclic systems [6]. The aim of this present work focuses on the synthesis of *N*-heterocyclic compounds, namely the synthesis of substituted isoxazole and triazole derivatives, since an important number of compounds containing the isoxazole and the triazole scaffold are known to exhibit a variety of biological activities in the pharmaceutical [16] and medicinal areas [17]. Representative examples of synthetic drugs incorporating these motifs are Tazobactam **I** [18,19,20,21,22], Rufinamide **II** [23,24,25], phenylisoxazole derivatives **III** [26,27], and Bextra (Valdecoxib) **IV** [28,29,30].

**Figure 1:**
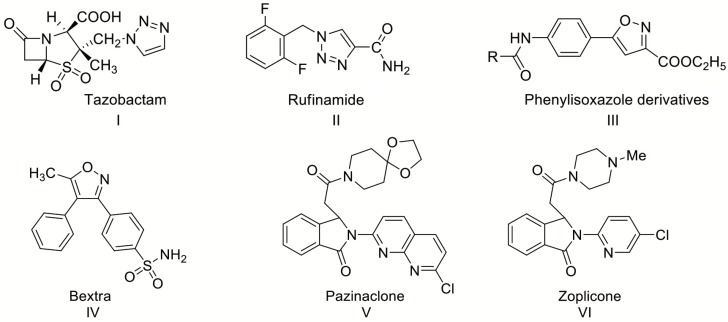
Structures of isoxazoles, triazoles and 3-substituted dihydroisoindolin-1-ones exhibiting biological or pharmaceutical activities.

On the other hand, the dihydroisoindolin-1-one ring system is present in numerous synthetic and natural compounds, which exhibit interesting biological properties. For example, 3-substituted dihydroisoindolin-1-ones such as Pazinaclone **V** [31] and Zoplicone **VI** [32,33,34] possess a pharmaceutical profile similar to that of the benzodiazepines [17] (sedatives, hypnotics) and have been commercialized as anxiolytics (Figure 1). Dihydroisoindolin-1-one derivatives have been extensively studied, but to the best of our knowledge there are no reports on dihydroisoindolin-1-ones incorporating an isoxazole or triazole moiety.

A classical route for the preparation of isoxazole or triazole is the reaction of alkynes with nitrile oxides and aryl azides, respectively [35,36,37]. In this context and in connection with our current research interest in the preparation of biologically relevant nitrogenated and oxygenated compounds by 1,3-dipolar cycloaddition on unsaturated systems with several 1,3-dipoles [38,39] we wish to describe in this paper an efficient synthesis of a series of novel 3,5-disubstituted isoxazoles and 1,4-disubstituted 1,2,3-triazoles by regioselective reaction of arylnitrile oxides **1a**–**d** and azides **2a**–**d** with the dihydroisoindolin-1-one-derived terminal alkyne **3**. For comparison, we have performed these 1,3-dipolar cycloadditions under non-catalyzed thermal activation in toluene or alternatively in the presence of the simple and inexpensive catalysts CuI and Ag_2_CO_3_. As well established for many other click reactions [40] catalyzed by Cu(I) salts, we obtained the best results using CuI as catalyst.

## 2. Results and Discussion

**Scheme 1:**
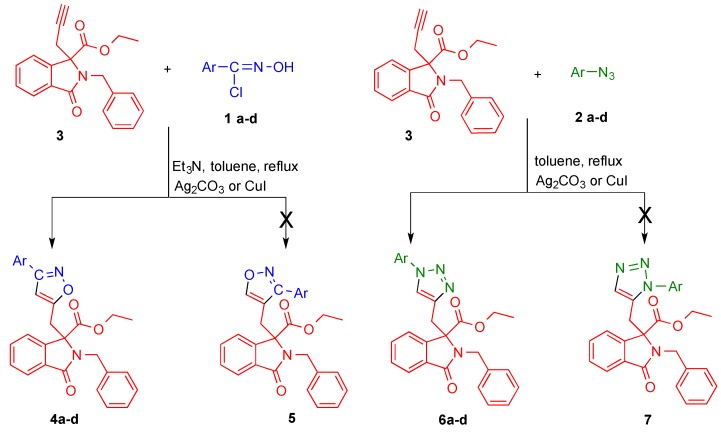
[3+2] Cycloaddition of dihydroisoindolin-1-one **3** with arylnitrile oxides **1** and azides **2**.

As previously described by our group [41] the dipolarophile **3** was prepared through an efficient four-step procedure starting from commercially available homophthalic acid, affording the desired bicyclic acetylenic lactam **3** in high yield. We have then examined the 1,3-dipolar cycloaddition reactions of propargyl-substituted dihydroisoindolin-1-one **3** as dipolarophile across aryl nitrile oxides **1a**–**d** (Ar: a: C_6_H_5_, b: *p*-MeC_6_H_4_, c: *p*-OMeC_6_H_4_, d: *p*-ClC_6_H_4_), generated *in situ* from aromatic oximes precursors [42] and azides [43] **2a**–**d** (Ar: a: C_6_H_5_, b: *p*-MeC_6_H_4_, c: *p*-MeOC_6_H_4_, d: *p*-ClC_6_H_4_), under conventional conditions (without catalyst) according to Scheme 1.

To find suitable reaction conditions, we conducted first the cycloaddition reaction in various solvents such as dichloromethane, acetonitrile, toluene and DMF both at room temperature and under reflux. Without catalyst, the targeted cycloadducts were only obtained in refluxing toluene, albeit in moderate amounts. The examination of the TLC of the reactions mixtures indicated the presence of only one product, which, after classical workup, was identified as cycloadducts **4** or **6** resulting from the thermal cycloaddition of the acetylene function and the 1,3-dipoles in accordance with the literature [44,45,46]. After optimization of the reactions conditions, we then performed all the cycloaddition reactions in presence of Ag_2_CO_3_ (10 mol %) using the same protocol given above.

Table 1 reveals that the silver-catalyzed reaction allowed us to obtain the corresponding isoxazoles **4a**–**d** and triazoles **6a**–**d** within a considerably shorter reaction time (24 h) in improved isolated yields (50%–73%) as colorless or yellowish solids. It is worth noting that, as far as we know, this is the first report on an efficient Ag_2_CO_3_-catalyzed 1,3-dipolar cycloaddition with a propargyl-substituted dihydroisoindolin-1-one as dipolarophile. The reaction times could be further shortened to only 6–8 h using 10 mol % of CuI as catalyst. Moreover, all [3+2] cycloadditions involving dipolarophile **3** and arylnitrile oxides **1a**–**d** or azides **2a**–**d** furnished the desired isoxazoles **4a**–**d** or triazoles **6a**–**d** (Ar: a: C_6_H_5_, b: *p*-MeC_6_H_4_, c: *p*-OMeC_6_H_4_, d: *p*-ClC_6_H_4_) in even superior yields (63%–89%) compared with the Ag_2_CO_3_-catalyzed syntheses.

**Figure 2:**
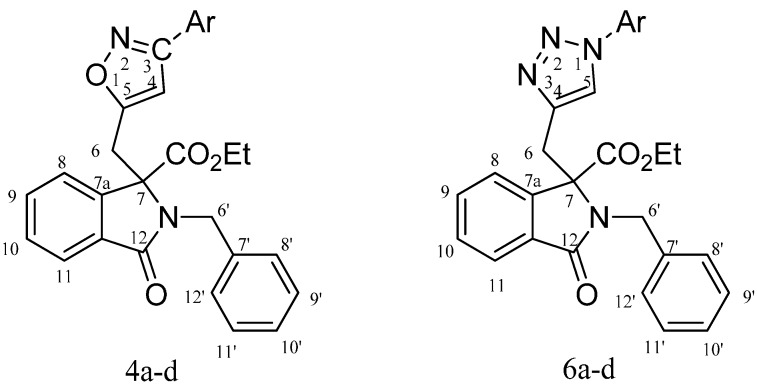
Numbering scheme of the isoxazole **4a**–**d** and 1,4-disubstituted 1,2,3-triazoles **6a**–**d**.

The structures of adduct **4a**–**d** and **6a**–**d** were confirmed through X-ray structure elucidation, or by XH correlation and HMBC spectra. It is noteworthy that, independently of the reaction temperature and catalyst ratio, TLC and NMR analysis indicated that the reaction seems to be highly regioselective. In all the cycloaddition tests, exclusively 3,5-disubstituted isoxazoles **4a**–**d** and 1,4-disubstituted 1,2,3-triazoles **6a**–**d** products were obtained (Figure 2).

### 2.1. Spectroscopic and Crystallographic Characterization of Isoxazole **4a**

**Figure 3:**
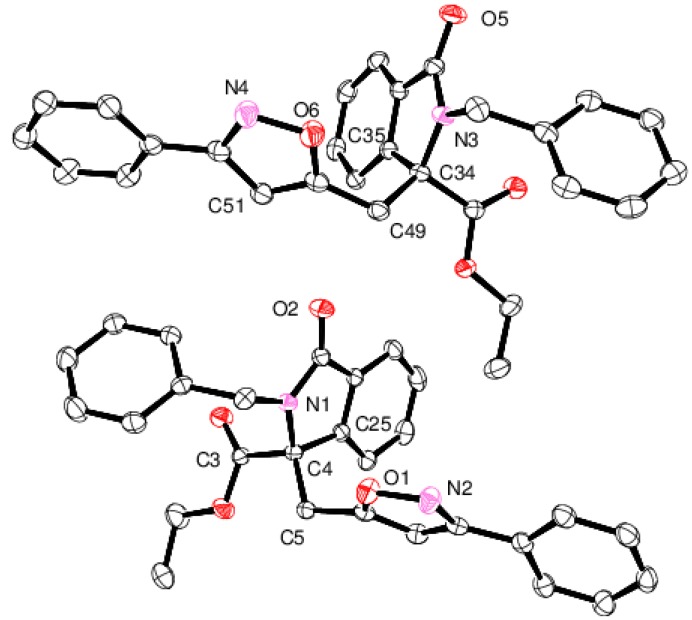
Content of the asymmetric unit in the racemic crystal of **4a** (P2_1_/c space group) showing the presence of both *S* (over C34 atom) and *R* (over C4 atom) enantiomers. Hydrogen atoms have been omitted for clarity. Thermal ellipsoids are plotted at 50% probability level. Selected bond lengths (Å) and angles (°): C4–C5 1.541(2), C4–N1 1.469(2), C4–C3 1.556(2), C4–C25 1.521(2), O1–N2 1.416(2), C34–C33 1.558(2), C34-N3 1.465(2), C34–C35 1.521(2), C34–C49 1.538(2), N4–O6 1.416(2); N1–C4–C25 101.4(1), N1–C4–C5 113.4(1), N1–C4–C3 109.1(1), C25–C4–C3 106.7(1), C25–C4–C5 113.7(1), C3–C4–C5 111.9(1), N3–C34–C35 101.5(1), N3–C34–C49 113.6(1), N3–C34–C33 108.9(1), C35–C34–C33 106.5(1), C35–C34–C49 113.9(1), C49-C34–C33 111.8(1).

The analytical and spectroscopic data are in agreement with the proposed structures illustrated in Scheme 1. The IR spectrum of compound **4a** (Ar = C_6_H_5_) contains an absorption band at ν_max_ = 1607 cm^−1^ characteristic of the isoxazole C=N group. The other absorptions at ν_max_ = 1744 and 1717 cm^−1^ are attributed to the ester C=O and lactam C=O stretching vibrations. The regiochemistry of this adduct was deduced from the ^1^H-NMR spectrum, which display the resonance of the isoxazole proton (4-H) as a singlet at δ = 5.18 ppm. This value of the chemical shift clearly confirms the regiochemistry of the cycloaddition and is in line with the values reported by Fokin, Sharpless [47,48,49] and our previous work; *i.e.*, the oxygen atom is bonded to more substituted carbon of an unsymmetrical double or triple bond. In the case of the hypothetical reverse regioisomer **5a**, one should expect a chemical shift value for the 5-H proton higher than 6 ppm due to the proximity of the isoxazolinic oxygen atom [50]. The two diastereotopic 6-H protons appear as two doublets at δ = 3.78 and 3.84 ppm with a ^2^*J* coupling constant of 15.3 Hz. No allylic coupling with 4-H was noticed. The second set of doublets at 4.74 (d, 1H, *J* = 15.2 Hz) and 4.85 ppm (d, 1H, *J* = 15.2 Hz) is attributed to the methylenic protons 6′-H, which are non-equivalent due to hindered rotation. Particularly characteristic are the ^13^C-NMR isoxazole ring resonances with three peaks at δ = 101.0, δ = 161.9 ppm and δ = 166.3 ppm for the isoxazole carbons 4-C, 5-C and 3-C. The carbon 7-C resonates at δ = 70.2 ppm and the two secondary carbons 6-C and 6′-C are observed at δ = 62.6 and 44.9 ppm, in accordance with the data reported by Fokin and Sharpless [47,48,49]. A further proof of our structural assignment stems from an X-ray structure determination [50] performed on **4a**, confirming unambiguously the stereochemistry of the product. Two independent molecules are present in the asymmetric unit of the P2_1_/c space group (Figure 3). The intrinsic racemic property of this space group (presence of improper symmetry operations: inversion and reflections) doesn’t need the presence of two independent molecules in the asymmetric unit for the overall racemic nature of the crystal. Such a presence of two chiral molecules in the asymmetric unit of the centrosymmetric space group is not a common feature of racemic crystals of chiral molecules. It is rather an indication of the low energy barrier upon S/R isomerization. Such a kind of isomerization in **4a** may occur during the crystallization process, but it may be also expected that both the relative *S* and *R* chiralities centered on the C4 and C34 atoms in two independent molecules should be already present in their solution before crystallization. The metric parameters (bond lengths and angles) observed for both isomers are found in the expected ranges and do not need further comments (Figure 3).

**Figure 4:**
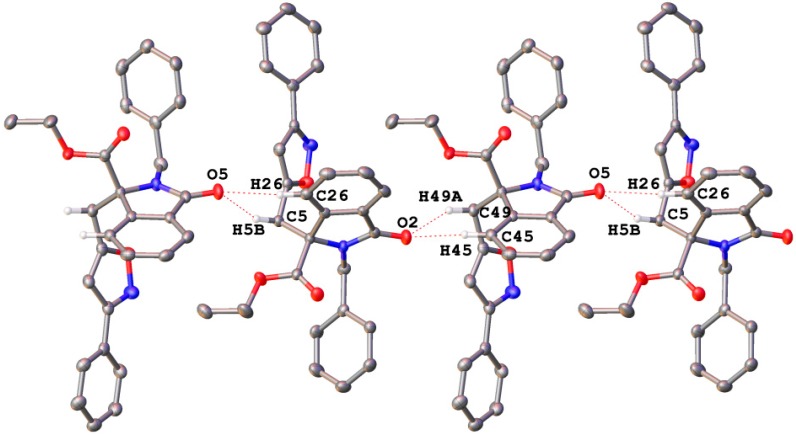
1D Chain structure of **4a** build through weak O···H–C hydrogen bonds.

There is a kind of intermolecular hydrogen bonds in the structure of **4a** consisting of weak C-H···O interactions leading to the formation of 1D arrays running along the *x* direction in the *xz* plane of the crystal lattice. Four associated molecules are shown in Figure 4. The two central ones are present in the asymmetric unit and the two lateral ones stem from the neighboring asymmetric units. The *intra* asymmetric unit (central) O···H–C (O2···C45/C49) distances are 3.253 and 3.302 Å, respectively. The O···H–C (O5···C5/C26) contacts within the *inter*-asymmetric unit (external) fall in the similar range of 3.213 and 3.304 Å.

### 2.2. Spectroscopic Characterization of Triazole **6a**

**Figure 5:**
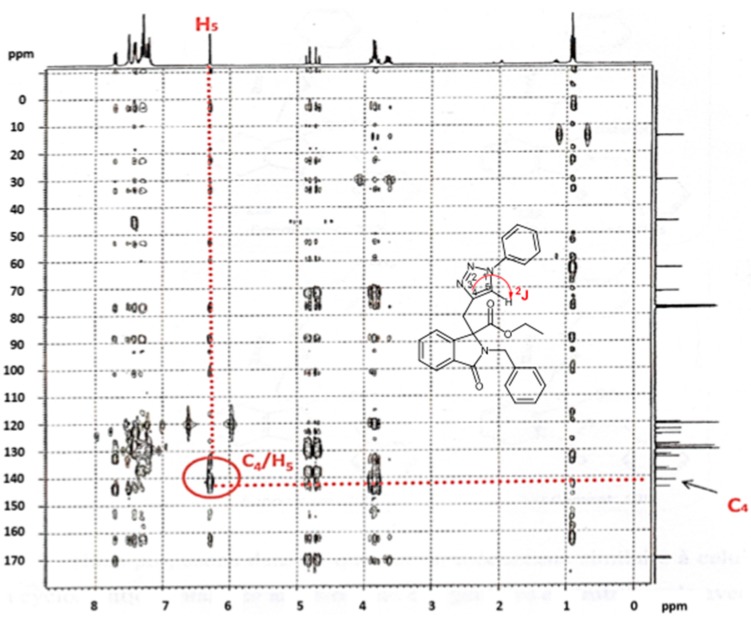
HMBC correlation spectrum of **6a**.

**Figure 6:**
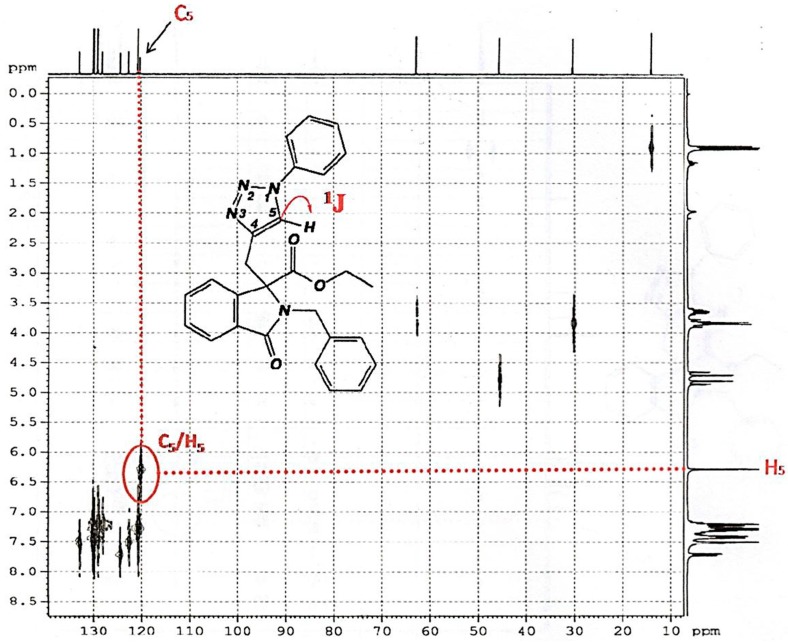
XH correlation spectrum of **6a**.

The IR spectrum of **6a** shows C=O absorptions bands at 1738 and 1701 cm^−1^ due to the ester and lactam carbonyls. The combination of ^1^H- and ^13^C-NMR spectroscopy allows one to deduce in an unambiguously manner the exclusive formation of triazolic regioisomers **6** as exemplified for **6a**. The characteristic signal of triazole proton 5-H appears as a singlet at δ = 6.34 ppm. Moreover, the presence of the ^2^*J* coupling constant of 15.2 Hz between the two diastereotopic protons 6-H at δ = 3.66–3.76 and 3.85–3.96 ppm on the one hand and the non-equivalent methylene protons 6′-H at δ = 4.75 and 4.90 ppm on the other hand as observed in the case of the cycloaddition of propargyl-substituted dihydroisoindolin-1-one **3** with arylnitrile oxides **1a**–**d** corroborating the structural assignment. An additional proof concerning the regiochemistry of the copper-catalyzed [3+2] cycloaddition reaction is provided by the ^13^C-NMR data. The chemicals shifts of the carbon atom 5-C at δ = 120.0 ppm and that of carbon 4-C at δ = 143.8 ppm are characteristic for 1,4-disubstituted-1*H*-1,2,3-triazoles [47,48,49,50,51]. The C atom 4-C of the hypothetical isomeric compound **7a** should appear at around 133 ppm [51]. Formation of these regioisomeric 1,5-disubstituted-1*H*-1,2,3-triazoles has been reported to occur by Ru-catalyzed cycloaddition [52,53], but we are not aware of copper-catalyzed reactions leading to these isomers. The carbon 7-C resonates at δ = 71.5 ppm, and the secondary carbon 6′-Cat δ = 62.7 ppm is downfield-shifted due to of deshielding effect of the nitrogen atom. The XH correlation spectrum of **6a** reveals a ^1^*J* correlation between the carbon 5-C and the 5-H proton, characteristic of a triazole cycle. The structural assignment of **6a** was furthermore confirmed by the analysis of its HMBC spectrum, where a ^2^*J* correlation between the proton 5-H and its adjacent carbon 4-C has been established (Figure 5 and Figure 6). The similarity of the spectroscopic data of derivatives **6b**, **6c** and **6d** with those of **6a** allows to conclude that all compounds of series **6** are isostructural, regardless of the electron-withdrawing or electron-donating propensity of the substituent at the *para*-position of the aryl group of azides **2a**–**d**.

## 3. Experimental Section

### 3.1. General Information

Reactions were carried out under an atmosphere of dry N_2_. Solvents were purified by standard methods and freshly distilled under nitrogen and dried before use, toluene was distilled under sodium. All melting points were measured on a Kofler bank. The IR infrared spectra were recorded spectra from KBr on a FT-IR Paragon 1000 IR spectrometer (Perkin-Elmer, Akron, Ohio, OH, USA) and only structurally significant bands are reported. Materials: solid thin-layer chromatography (TLC); TLC plates (silica gel 60 F_254_ 0.2 mm 20 cm × 20 cm or with aluminum plates (0.20 mm) precoated with fluorescent silica gel, Merck); substances were detected using UV light at 254 nm. TLC was performed using EtOAc/cyclohexane as eluent. Reaction components were then visualized under UV light and dipped in iodine or a Dragendorff solution. All reactions were performed under an inert atmosphere. NMR spectra were recorded with a Bruker AC 300 spectrometer (Bruker Spectrospin, Rheinstetten, Germany) operating at 300 MHz for ^1^H and 75.5 MHz for ^13^C using TMS as the internal standard (0.00 ppm) in CDCl_3_ as solvent. Chemical shifts were reported in ppm downfield from TMS.

### 3.2. Typical Procedure of the Cycloaddition Reaction of Arylnitrile Oxides **1a**–**d** and Ethyl-3-alkyldiynyl-phtalimidine-3-carboxylate (**3**)

To a mixture of dipolarophile **3** (0.9 mmol) and arylnitrile oxides precursors **1a**–**d** (0.9 mmol) in 10 mL of degassed toluene was added triethylamine (0.9 mmol) and CuI or Ag_2_CO_3_ (10 mol %). The reaction mixture was refluxed for 6–8 h. After filtration of triethylamine hydrochloride, the filtrate was concentrated under reduced pressure. The corresponding cycloadducts was obtained by crystallization in a minimum of EtOH.

*Ethyl 2-benzyl-3-oxo-1-((3-phenyl)isoxazol-5-yl)methyl)-2*,*3-dihydro-1H-isoindole-1-carboxylate* (**4a**). Yield 85%; colorless solid; mp 147–149 °C; IR (ν, cm^−1^ KBr) 3031, 1744, 1717, 1607, 1236; ^1^H-NMR: δ 0.97 (t, 3H, *J* = 7.1 Hz), 3.68–3.75 (d, 1H, *J* = 15.3 Hz), 3.84 (q, 2H, *J* = 7.1 Hz), 3.87–3.97 (d, 1H, *J* = 15.3 Hz), 4.74 (d, 1H, *J* = 15.2 Hz), 4.85 (d, 1H, *J* = 15.2 Hz), 5.18 (s, 1H), 7.24–7.84 (m, aromatic H) ppm; ^13^C-NMR: δ 13.6 (CH_3_), 30.7 (CH_2_), 44.9 (CH_2_), 62.6 (CH_2_), 70.6 (Cq), 101.0 (CH_isox_), 121.7 (CH), 124.2 (CH), 126.6 (2CH), 127.8 (CH), 128.6 (CH), 128.7 (2CH), 129.1 (2CH), 129.7 (CH), 129.9 (CH), 131.4 (CH), 132.4 (CH), 136.9 (CH), 142.9 (CH), 161.9 (C_isox_), 166.3 (C_isox_), 169.2 (CO), 169.5 (CO). Anal. Calcd for C_28_H_24_N_2_O_4_ (452.17): C, 74.32; H, 5.35; N, 6.19. Found: C, 74.50; H, 5.20; N, 6.10.

*Ethyl 2-Benzyl-3-oxo-1-((3-(4-methylphenyl)isoxazol-5-yl)methyl)-2,3-dihydro-1H-isoindole-1-carboxylate* (**4b**). Yield 63%; colorless solid; mp 115–117 °C; IR (ν, cm^−1^ KBr) 3034, 1733, 1717, 1700, 1257; ^1^H-NMR: δ 0.99 (t, 3H, *J* = 7.2 Hz), 2.36 (s, 3H), 3.70–3.80 (d, 1H, *J* = 15.3 Hz), 3.86 (q, 2H, *J* = 7.2 Hz), 3.91–3.99 (d, 1H, *J* = 15.3 Hz), 4.76 (d, 1H, *J* = 15.2 Hz), 4.86 (d, 1H, *J* = 15.2 Hz), 5.28 (s, 1H), 7.17–7.86 (m, aromatic H) ppm; ^13^C-NMR: δ 13.9 (CH_3_), 21.7 (CH_3_), 31.1 (CH_2_), 45.2 (CH_2_), 62.9 (CH_2_), 70.6 (Cq), 101.2 (CH_isox_), 122.0 (CH), 124.5 (CH), 126.2 (CH), 126.8 (2CH), 128.1 (CH), 128.9 (2CH), 129.5 (2CH), 130.0 (CH), 131.8 (CH), 132.7 (CH), 137.2 (CH), 140.3 (CH), 143.3 (CH), 162.3 (C_isox_), 166.5 (C_isox_), 169.6 (CO), 169.8 (CO). Anal. Calcd for C_29_H_26_N_2_O_4_ (466.20): C, 74.66; H, 5.62; N, 6.00. Found: C, 74.82; H, 5.74; N, 5.94

*Ethyl 2-Benzyl-3-oxo-1-((3-(4-methoxyphenyl)isoxazol-5-yl)methyl)-2*,*3-dihydro-1H-isoindole-1-carboxylate* (**4c**). Yield 75%; yellow solid; mp 169–171 °C; IR (ν, cm^−1^ KBr) 3030,1734, 1692, 1611, 1250; ^1^H-NMR: δ 0.98 (t, 3H, *J* = 7.1 Hz), 3.64–3.75 (d, 1H, *J* = 15.3 Hz), 3.80 (s, 3H), 3.84 (q, 2H, *J* = 7.1 Hz), 3.85–3.90 (d, 1H, *J* = 15.3 Hz), 3.92–3.98 (d, 1H, *J* = 15.2 Hz), 4.71–4.87 (d, 1H, *J* = 15.2 Hz), 5.18 (s, 1H); 7.27–7.86 (m, aromatic H) ppm; ^13^C-NMR: δ 13.6 (CH_3_), 30.7 (CH_2_), 44.8 (CH_2_), 62.6 (CH_2_), 70.2 (Cq), 100.8 (CH_isox_), 121.6 (CH), 124.1 (CH), 127.1 (CH), 127.7 (CH), 127.8 (2CH), 128.5 (2CH), 128.9 (2CH), 129.1 (2CH), 129.7 (CH), 131.3 (CH), 132.4 (CH), 135.8 (CH), 136.8 (CH), 142.8 (CH), 160.9 (C_isox_), 166.6 (C_isox_), 169.2 (CO), 169.4 (CO). Anal. Calcd for C_29_H_26_N_2_O_5_ (482.18): C, 72.18; H, 5.43; N, 5.81. Found: C, 72.08; H, 5.35; N, 5.72.

*Ethyl 2-Benzyl-3-oxo-1-((3-(4-chlorophenyl)isoxazol-5-yl)methyl)-2*,*3-dihydro-1H-isoindole-1-carboxylate* (**4d**). Yield 67%; colorless solid; mp 134–136 °C; IR (ν, cm^−1^ KBr) 3066, 1734, 1695, 1610, 1263; ^1^H-NMR: δ 0.98 (t, 3H, *J* = 7.1Hz), 3.70–3.79 (d, 1H, *J* = 15.3 Hz), 3.84 (q, 2H, *J* = 7.1 Hz), 3.92–3.96 (d, 1H, *J* = 15.3 Hz), 4.71 (d, 1H, *J* = 15.1 Hz), 4.87 (d, 1H, *J* = 15.1 Hz), 5.18 (s, 1H), 7.27–7.86 (m, aromatic H) ppm; ^13^C-NMR: δ 13.6 (CH_3_), 30.7 (CH_2_), 44.8 (CH_2_), 62.6 (CH_2_), 70.2 (Cq), 100.8 (CH_isox_), 121.6 (CH), 124.1 (CH), 127.1 (CH), 127.7 (CH), 127.8 (2CH), 128.5 (2CH), 128.9 (2CH), 129.1 (2CH), 129.7 (CH), 131.3 (CH), 132.4 (CH), 135.8 (CH), 136.8 (CH), 142.8 (CH), 160.9 (C_isox_), 166.6 (C_isox_), 169.2 (CO), 169.4 (CO). Anal. Calcd for C_28_H_23_ClN_2_O_4_ (486.13): C, 69.06; H, 4.76; Cl, 7.28; N, 5.75. Found: C, 68.98; H, 4.94; Cl, 7.32; N, 5.57.

### 3.3. Typical Procedure of the Cycloaddition Reaction of Azides **2a**–**d** and Ethyl 3-Alkyldiynylphtalimidine-3-carboxylate (**3**)

A mixture of dipolarophile **3** (0.9 mmol), azides **2a**–**d** (0.9 mmol) and CuI or Ag_2_CO_3_ (10 mol %) in dry toluene (10 mL) was stirred under argon atmosphere at 110 °C. after the completion of the reaction indicated by TLC analysis, solvent was evaporated under reduced pressure and the crude mixture was purified by crystallization in a minimum of EtOH solution.

*Ethyl 2-Benzyl-3-oxo-1-((1-phenyl-1H-1*,*2*,*3-triazol-4-yl)methyl)-2*,*3-dihydro-1H-isoindole-1-carboxylate* (**6a**). Yield 89%; yellow solid; mp 145–147 °C; IR (ν, cm^−1^ KBr) 3036, 1738, 1701, 1242 ; ^1^H-NMR: δ 0.97 (t, 3H, *J* = 7.1 Hz), 3.66–3.76 (d, 1H, *J* = 15.2 Hz), 3.85–3.96 (d, 1H, *J* = 15.2 Hz), 3.91 (q, 2H, *J* = 7.1 Hz), 4.75 (d, 1H, *J* = 15.4 Hz), 4.90 (d, 1H, *J* = 15.4 Hz), 6.34 (s, 1H**_triaz_**), 7.22–7.77 (m, aromatic H) ppm; ^13^C-NMR: δ 14.0 (CH_3_), 30.2 (CH_2_), 45.4 (CH_2_), 62.7 (CH_2_), 71.5 (C**_q_**), 120.0 (CH**_triaz_**), 120.5 (2CH), 122.4 (CH), 124.2 (CH), 128.0 (CH), 128.9 (CH), 129.7 (2CH), 129.8 (CH), 129.9(2CH), 131.9 (CH), 132.6 (CH), 137.0 (CH), 141.3 (CH), 143.8 (C_triaz_), 169.8 (CO), 170.2 (CO). Anal. Calcd for C_27_H_24_N_4_O_3_ (452.18): C, 71.67; H, 5.35; N, 12.38. Found: C, 71.80; H, 5.42; N, 12.42.

*Ethyl 2-Benzyl-3-oxo-1-((1-(4-methylphenyl)-1H-1*,*2*,*3-triazol-4-yl)methyl)-2*,*3-dihydro-1H-isoindole-1-carboxylate* (**6b**). Yield 75%; colorless solid; mp 146–148 °C; IR (ν, cm^−1^ KBr) 3035, 1735, 1701, 1246 ; ^1^H-NMR: δ 0.90 (t, 3H, *J* = 7.1 Hz), 2.29 (s, 3H), 3.61–3.64 (d, 1H, *J* = 15.2 Hz), 3.83 (q, 2H, *J* = 7.1 Hz), 3.80–3.82 (d, 1H, *J* = 15.2 Hz), 4.69 (d, 1H, *J* = 15.4 Hz), 4.82 (d, 1H, *J* = 15.4 Hz), 6.24 (s, 1H), 7.10–7.71 (m, aromatic H) ppm ; ^13^C-NMR: δ 12.5 (CH_3_), 20.0 (CH_3_), 28.8 (CH_2_), 44.0 (CH_2_), 61.3 (CH_2_), 70.0 (C**_q_**), 118.6 (CH**_triaz_**), 119.0 (2CH), 121.0 (CH), 122.8 (CH), 126.6 (CH), 127.5 (2CH), 128.2 (2CH), 128.3 (CH), 128.9 (2CH), 130.5 (CH), 131.2 (CH), 133.3 (CH), 136.3 (CH), 137.5 (CH), 139.7 (CH), 142.4 (C**_triaz_**), 168.3 (CO), 168.8 (CO). Anal. Calcd for C_28_H_26_N_4_O_3_ (466.20): C, 72.09; H, 5.62; N, 12.01. Found: C, 71.98; H, 5.80; N, 12.09.

*Ethyl 2-Benzyl-3-oxo-1-((1-(4-methoxyphenyl)-1H-1,2,3-triazol-4-yl)methyl)-2,3-dihydro-1H-isoindole-1-carboxylate* (**6c**). Yield 85%; pink solid; mp 165–167 °C; IR (ν, cm^−1^ KBr) 3033, 1735, 1690, 1254; ^1^H-NMR: δ 0.89 (t, 3H, *J* = 7.1Hz), 3.74 (s, 3H), 3.77–3.82 (d, 1H, *J* = 15.1 Hz), 3.83 (q, 2H, *J* = 7.1 Hz), 4.80 (d, 1H, *J* = 15.1 Hz), 4.85 (d, 1H , *J* = 15.4 Hz), 4.92 (d, 1H, *J* = 15.4 Hz), 6.20 (s, 1H), 6.80–7.71 (m, aromatic H) ppm; ^13^C-NMR: δ 12.5 (CH_3_), 28.8 (CH_2_), 44.0 (CH_2_), 54.5 (CH_3_), 61.3 (CH_2_), 70.0 (Cq), 113.4 (2CH), 118.7 (CH_triaz_), 120.7 (2CH), 121.0 (CH), 122.8 (CH), 126.5 (CH), 127.5 (2CH), 128.2 (2CH), 128.3 (CH), 129.0 (CH), 130.5 (CH), 131.2 (CH), 136.3 (CH), 139.6 (CH), 142.4 (C_triaz_), 168.3 (CO), 168.8 (CO). Anal. Calcd for C_28_H_26_N_4_O_4_ (482.20): C, 69.70; H, 5.43; N, 11.61. Found: C, 69.85; H, 5.37; N, 11.57.

*Ethyl 2-Benzyl-3-oxo-1-((1-(4-chlorophenyl)-1H-1*,*2*,*3-triazol-4-yl)methyl)-2*,*3-dihydro-1H-isoindole-1-carboxylate* (**6d**). Yield 67%; colorless solid; mp 154–156 °C; IR (ν, cm^−1^ KBr) 3056, 1725, 1697, 1245; ^1^H-NMR: δ 0.93 (t, 3H, *J* = 7.1 Hz), 3.66–3.68 (d, 1H, *J* = 15.2 Hz), 3.83 (q, 2H, *J* = 7,1 Hz), 3.90 (d, 1H, *J* = 15.2 Hz), 4.63 (d, 1H, *J* = 15.2 Hz), 4.86 (d, 1H, *J* = 15.2 Hz), 6.18 (s, 1H), 7.19–7.70 (m, aromatic H) ppm; ^13^C-NMR: δ 12.6 (CH_3_), 28.7 (CH_2_), 44.1 (CH_2_), 61.4 (CH_2_), 70.1 (Cq), 118.6 (CH_triaz_), 120.2 (2CH), 121.0 (CH), 122.8 (CH), 126.6 (CH),127.5 (2CH), 128.3 (2CH), 128.4 (CH), 128.6 (2CH), 130.4 (Cq), 131.3 (C H), 133.1 (CH), 134.0 (CH), 136.4 (CH), 140.1 (CH), 142.3 (C_triaz_), 168.4 (CO), 168.8 (CO). Anal. Calcd for C_27_H_23_ClN_4_O_3_ (486.15): C, 66.60; H, 4.76; Cl, 7.28; N, 11.51. Found: C, 66.83; H, 4.68; Cl, 7.20; N, 11.70.

### 3.4. X-ray Diffraction Structure Analysis of **4a**

A colorless crystal of **4a** has been mounted on a Apex II diffractometer (Bruker Nonius Kappa, Karlsruhe, Germany) and the intensity data have been collected at 115 K with Mo Kα radiation of λ = 0.71073 Å. These data were further treated with Denzo and Scalpack programs [54]. The model of the structure has been solved by direct methods with SIR92 [55] and refined with SHELXL-97 [56]. All non-hydrogen atoms were refined with anisotropic temperature factors. All H atoms were placed in calculated positions and refined as riding on the heavy atoms bearing them. The resolution of the structure revealed the presence of two enantiomer molecules in the asymmetric unit of P2_1_/c space group.

Crystallographic data have been deposited with the Cambridge Crystallographic Data Center: deposition number CCDC 1405500 contains detailed crystallographic data for this publication. These data may be obtained free of charge from the Cambridge Crystallographic Data Center through www.ccdc.cam.ac.uk/data_request/cif.

## 4. Conclusions

We have developed a new and efficient method for the synthesis of novel isoxazole and 1,2,3-triazole-substituted dihydroisoindolin-1-ones. The process involves regiospecific [3+2] cycloaddition between a propargylic alkyne and arylnitrile oxides or azides using Ag_2_CO_3_ or CuI as simple commercially available catalysts in toluene at 110 °C. The electron-withdrawing or electron-donating propensity of the substituent at the *para*-position of the aryl group of arylnitrile oxides **1** or azides **2** have no impact on the outcome of the reaction; future studies will show whether a substitution at the *ortho*-position may influence the stereochemistry of these cycloaddition across the acetylenic dipolarophile **3**. The biological activity and inhibitory effect of such analogues is under examination, as well as applications in analogous natural product syntheses.

## Figures and Tables

**Table 1:** Formation of isoxazoles **4a**–**d** and triazoles **6a**–**d** under conventional and catalytic conditions.

Products ^a^	Conventional Conditions		Ag_2_CO_3_-Catalyzed Conditions ^b^		CuI-Catalyzed Conditions ^b^	
	Time (h)	Yield (%)	Time (h)	Yield (%)	Time (h)	Yield (%)
**4a**	48	57	24	73	6	85
**4b**	120	28	24	58	6	63
**4c**	48	59	24	70	6	75
**4d**	72	47	24	50	8	63
**6a**	48	62	24	73	6	89
**6b**	72	48	24	58	6	75
**6c**	48	59	24	70	6	85
**6d**	48	42	24	53	6	67

^a^ all reactions were carried out using toluene at reflux under Argon; ^b^ the reaction was carried out using 10 mol % of catalyst in presence of Et_3_N.
